# Education in Robotic Hernia Surgery—Current Situation

**DOI:** 10.3389/jaws.2023.12418

**Published:** 2023-11-30

**Authors:** Sergio Roll, Eduardo Rullo Maranhão Dias

**Affiliations:** ^1^ Department of Surgery, School of Medical Sciences of Santa Casa of São Paulo, São Paulo, Brazil; ^2^ Center of Abdominal Wall Surgery, Division of General Surgery, Santa Casa of São Paulo Hospital, School of Medical Sciences of Santa Casa of São Paulo, São Paulo, Brazil; ^3^ The German Hospital Oswaldo Cruz Hernia Center, São Paulo, Brazil; ^4^ Digestive Surgery, Abdominal Wall Division, Santa Casa de São Paulo, São Paulo, Brazil

**Keywords:** education, robotic surgery, hernia surgery, artificial intelligence, virtual reality

## Introduction

I would like to start this article by looking back at the time when I graduated from medical school in 1982 and began my surgical training during my medical residency. At that time, the practice of medicine was more of an art, grounded in the scientific knowledge we acquired through learning. There was no formal system of competency-based assessment or appraisal or annual review, and at the end of my specialist training, a simple system of accreditation determined its successful completion.

Skills were acquired through a somewhat informal but time-honored approach known as “see one, do one, teach one,” which can be traced back to the pioneering efforts of William Stewart Halsted, who introduced this method within the surgical residency program at Johns Hopkins University in the late 19th century. Halsted’s students would begin training by watching surgical techniques performed, thereby gaining a model for their own actions. Halsted also developed a core set of principles advocating delicate tissue manipulation, preservation of blood supply, meticulous hemostasis, elimination of dead space, careful tissue approximation, and tension-free closure.

The advent of better surgical techniques and technologies has gradually enabled the better achievement of Halsted’s principles, and medical training has become highly structured and comprehensively assessed, and achieving competence is now seen as the measure of medical attainment, which was not the case when I started my surgical training.

Here, I want to specifically consider the current situation in respect of the training of surgeons to use robotic surgery, a surgical method that was introduced in the early 2000s and is a significant technological advance. Its introduction has a number of advantages over traditional surgery, including improved visualization of the surgical field, enabling greater precision and accuracy in movements, leading to better outcomes. Despite the initial cost, which reduces accessibility, when used efficiently and effectively improved outcomes can be achieved, which translate into increased cost-effectiveness. The development of new methods and technologies since the time of Halsted means that the education and training of surgeons can no longer be based purely on his principles, as was still by and large the case in 1982, but need to be enhanced using all the tools available today and include continuous assessment of competence.

In respect of training for robotic surgery, simulations can be used in various environments, ranging from laboratories with models designed for the practice of cutting, dissection, and suturing using real robotic instruments inside a “trainer box,” to surgeries on cadavers or animals. More recently, virtual reality (VR) has been used to create simulations to train surgeons in everything from basic tasks to complex operative steps. The use of VR is more suited to robotic rather than traditional surgery, as it is better able to realistically simulate the experience.

## Learning Is an Activity That Leads to a Sustained Change in Behavior

When discussing learning, it is important to remember the work done by Benjamin Bloom in 1948, who proposed the domains of learning. In a working group of university examiners, there was a discussion about the difficulty in exchanging assessment tools (e.g., multiple choice questions) as they used different definitions for the same terms, and it was agreed to create a taxonomy to facilitate communication between examiners. At the most basic level, this would allow the exchange of exam questions with standardized meanings. A group was formed to move forward with the project [1, 2] and they classified the work into three domains [1]:

• Cognitive (thinking): problem solving,
• Affective (feeling): engagement,
• Psychomotor (action): physical manipulation

It was agreed that the basis for the taxonomy would be a classification of the objectives of the educational process: educational objectives. Educational objectives define the specific behavioral changes expected of students from a given learning experience. Later in 2001, Anderson, Krathwohl and collaborators described a modification to Bloom’s taxonomy which addresses some of the problems encountered with operationalizing the original taxonomy [2].

## Robotics and AI

Artificial intelligence is constantly evolving and improving. Unlike in the past, when wisdom was associated with having the most information, perhaps from now on it will be defined by one’s ability to extract precise and accurate information, given that artificial intelligence stores data on a much larger scale and with greater precision than the human brain can.

In a survey conducted by Pew Research Center’s in 2014 about the digital life in 2025, it was reported that most respondents predicted that robotics and artificial intelligence would permeate broad segments of daily life by 2025, with enormous implications for a range of industries such as healthcare, transport and logistics, customer service and home maintenance [3]. In one of the interviewees the CEO of a software technology company, and an active participant in Internet standards development, responded, “Hopefully one of the areas where this will have most impact is the medical field—this is an area where there are high costs, a shortage of highly skilled people and a growing demand for advanced and complex services.”

Although artificial intelligence and robotic surgery are constantly evolving, there is still some resistance to their use, in part due to the complex nature of the interaction with human tissue. Thus, the full potential for interaction between intelligent systems, the surgeon and the patient has yet to be explored.

## Training in Robotic Hernia Surgery

### The Challenge

With the emergence and expansion of robotic surgery, several common general surgical procedures are now performed with robotic assistance [4]. The repair of abdominal wall hernias, due to their prevalence in the general population, is the most commonly performed surgery undertaken using robotic platforms among general and digestive system surgeries. There is thus a growing demand for robotically trained surgeons [5, 6]. The current challenge is to offer quality training to surgeons to ensure their proficiency in applying these new technologies [7, 8].

Given the increasing prevalence of robotic inguinal hernia repairs being performed around the world, it is imperative to consider the significant impact of surgeon training on both patient outcomes and healthcare costs. Developing an optimized training paradigm for credentialing and privileging surgeons is crucial as it will not only affect how we train aspiring robotics surgeons, but also influence the adoption of emerging new surgical technologies.

One of the primary challenges lies in accommodating surgeons who are not part of a medical residency or fellowship program, which typically offer long-term training in knowledge and development of surgical skills [9]. The proposed solution outlined here involves establishing a minimum training curriculum for performing robotic surgical procedures. The aim is to equip surgeons with the necessary knowledge and skills that allows them to reach a certain level of proficiency before performing surgical procedures on humans. The qualification process must ensure that those surgeons who receive credentials are able to effectively navigate the technical challenges of the learning curve and perform procedures safely and independently. Below, we describe in detail a training model for robotic surgery and a specific model for robotic hernia surgery.

### Fundamental Concepts of Robotic Surgery

I – Introduction to the robotics system [10]:
  1. Introduction to the robotic platform
  2. Product training (web-based with specific certification).
II – Theoretical and practical training in the use of the robotic platform (“in-service”) [10]:
  1. Preparation of the robotic platform in the operating room
  2. System set-up
  3. Docking
  4. Troubleshooting
  5. Theoretical and practical classroom situations using the platform in a surgical room (or simulation center).
III – Post system training (pre-clinical) – curriculum for the development of psychomotor skills [10–12]:
  Methods:
  1. Virtual Reality Simulation
  2. “Real” simulation
  3. Simulation in organic models [13, 14].
IV – Clinical training under supervision:
  Phases:
  1. Observation of cases in the operating room
  2. Participation as the assistant surgeon (bedside assistant)
  3. Perform robotic surgical procedures under supervision.
V – Post-training

Continuing education in respect of “advanced” procedures, the maintenance of robotic surgery skills, and the maintenance of privileges in robotics.

### Specific Training in Robotic Hernia Surgery

Robotic technology is being adopted for use in a wide array of general surgical cases; however, the transition to minimally invasive approaches may elicit a variety of procedure-specific challenges. The existing literature indicates that there is a relatively smooth transition from laparoscopic to robotic-assisted inguinal hernia repair with identical mean surgical times, similar outcomes, and no significant differences in recurrence rates [15]. However, transitioning from open to robotic inguinal repair may prove to be more difficult.

Surgeons starting a training pathway in respect of robot assisted abdominal wall surgery in their clinical practice will come from different backgrounds with respect to both exposure to abdominal wall surgery and robotic expertise. The baseline experience of the surgeon will, therefore, likely influence the learning trajectory they would need to follow to ensure a safe and efficient training pathway.

### Step-by-Step Proposal

• Phase I: Introduction to robotic technology and hernia surgery: understanding the anatomy of the inguinal region and the muscular composition of the abdominal wall as well as the techniques currently performed in inguinal and ventral incisional hernias
• Phase II: Training in robotic technology
• Phase III: Simulation-based training—the surgeons in training should be required to undertake this training and understand that even with previous experience in laparoscopy there is a learning curve in respect of robotic surgery. The repetition of a simulated tasks (under supervision) over a period of time leads to an improvement in results.
• Phase IV: Initial case series: the best cases for beginners are primary inguinal hernia and small ventral or umbilical hernias (intraperitoneal underlay mesh—IPUM)
• Phase V: Continued development and advanced training: surgeon-led training with a focus on the technical and anatomical aspects of abdominal wall repair procedures (robotic retrorectus ventral hernia repair (RRVHR) [16–20].

## Discussion

Robotic surgery training is constantly evolving, and there are many tools available to support didactic-pedagogical planning. Bloom et al. (1956) in their seminal work on the taxonomy of educational objectives classified them into three domains, namely, the cognitive, affective, and psychomotor. While all three domains have been widely discussed at different times and by different researchers, the cognitive domain, which pertains to intellectual skills and knowledge acquisition, such as remembering, understanding, and applying information, is the most widely known and used. Many educators rely on the theoretical assumptions of this domain to define objectives, strategies, and assessment systems in their educational planning [1, 2].

Different robotic platforms demand different educational protocols and planning. Currently, applications already exist that allow data to be managed in new ways to provide surgeons with real-time actionable insights to help them to improve their performance, reduce variability, and enhance long-term outcomes. The recording of surgical times, the number of assertive and unnecessary movements, and complications not only makes it possible to review and analyze information quickly so that the surgeon can focus on decision-making, but can also feed into big data systems, which are the primary prerequisite for developing autonomous robots [21].

Practicing in virtual reality simulators allows students to have visual and sensory exchanges that enable them to develop coordination skills for later practical application. A study by Wu et al. evaluated cognitive engagement during simulator training using electroencephalography, and by measuring eye movement and pupil diameter. The authors found that changes in cognitive and behavioral states predicted training outcomes with 72.5% accuracy, and that when the trainees were highly engaged in the virtual reality tasks, they tended to acquire the trained skill more reliably [22].

Current guidelines propose a minimum curriculum for the development of proficiency for performing robotic surgical procedures. The curriculum must integrate training and objective performance evaluation. In summary, the training will consist of a pre-clinical stage aimed at knowledge and adaptation to a specific robotic platform and the development of psychomotor skills based on surgical simulation. It may involve the use of organic models, animal or human cadavers or experimental animals, but this is not mandatory. The surgeon must then: 1) attend at least five operations, performed by a preceptor surgeon; 2) participate as an assistant surgeon (a bedside assistant) in at least 10 cases and 3) perform 10 operations under the supervision of a preceptor surgeon. The preceptor surgeon must be duly qualified in robotic surgery and have a minimum experience of 35–50 robotic procedures.

In abdominal wall surgery in particular, it is essential to customize the treatment for each patient’s specific condition, and there are several technical options available for surgical treatment. Thus, this is an area in which specialized training, with different learning curves for each procedure, is required [16].

In respect of the qualification, the current trend is for the final certification to be issued by the relevant medical societies rather than by the companies that own the robotic systems.

In conclusion, the surgeon who completes all the steps described above should be considered qualified in robotic surgery as a specialty.
